# Editorial: Multi-Omics Revolution in Microbial Cultural Heritage Conservation

**DOI:** 10.3389/fmicb.2021.720509

**Published:** 2021-08-30

**Authors:** Massimiliano Marvasi, Domenico Pangallo, Duccio Cavalieri, Fernando Poyatos-Jiménez

**Affiliations:** ^1^Department of Biology, University of Florence, Florence, Italy; ^2^Department of Microbial Ecology, Institute of Molecular Biology, Slovak Academy of Sciences, Bratislava, Slovakia; ^3^Department of Painting, Faculty of Fine Arts, University of Seville, Seville, Spain

**Keywords:** cultural heritages, MinION, metagenomics, Illumina, paper, parchment, stone consolidation

In recent decades the conservation of cultural heritage has been attracting increasing research interest from many different scientific disciplines. Lately, the co-integration of chemistry and physics with biological techniques has shaped our understanding of the living microbial communities on sites of cultural heritage (Marvasi et al., 2019; Pavlović et al., 2021). The study of microbial communities requires a combination of different techniques. Multi-omics techniques, where the prefix- omics includes genomics, transcriptomics, proteomics, metabolomics, represents a new frontier in conservation to better study the ecology, taxonomy, and interactions of the microbes colonizing cultural heritage items. This Research Topic brings together a number of examples, making connections relating to omics technology and showing how revolutionary the co-application of these techniques can be in the conservation of sites of cultural heritage.

This Research Topic discusses the very interesting concept of the definition of “bio-archive” for the determination of a core microbiome with the scope of monitoring, cataloging, and providing added-value to artworks (Piñar et al.). This concept has been applied to the study of some of Leonardo da Vinci's most emblematic drawings. This includes the famous Leonardo *autoritratto* (self-portrait). Results were achieved via Oxford Nanopore sequencing technology. The drawings' microbial bio-archive showed a relatively high contamination with human DNA and a surprising dominance of bacteria over fungi. The study also showed the presence of cellulose degraders, such as several members of the phyla Proteobacteria, Actinobacteria, and Firmicutes among bacteria, and fungi belonging to the classes Sordariomycetes and Eurotiomycetes. Interestingly, the microbial profile correlated with that of Rome or Turin were also identified, indicating the importance of environmental and storage conditions on the specific microbiota (Piñar et al.).

The application of -omics techniques has also found great applications in archaeology with a specific focus on stone materials. Two studies dealt with stone conservation and degradation. The first study evaluated the changes in bacterial diversity of severely degraded tuff stone and lime plaster at the archaeological Maya site of Copan (Honduras) after treatment with the patented sterile M-3P nutritional solution (Jroundi et al.). Illumina MiSeq technology showed that after treatment an enrichment of bacteria able to foster calcium carbonate biomineralization was achieved, and this supported stone consolidation. This is an excellent example of how bio-conservation treatment can safely and effectively be applied to temples, sculptures, and stuccos, and how metagenomics is an effective tool to assess the quality of the intervention (Jroundi et al.).

Another example on the protection of stone showed the biodegradation potential by microorganisms against the protective epoxy resin in the archaeological Ruins of Liangzhu City (Cina) (Sun et al.). In addition, the study reported a meta-taxonomical overview (*via* Illumina sequencing) of the bacterial and fungal microbial community (Sun et al.).

A valuable approach of integration among -omics approaches has been proposed by studying the biodeterioration dynamics of parchment. Such dynamics have been explored by adopting a multidisciplinary approach combining standard microbiological methods with high-throughput molecular, chemical, and physical techniques (Perini et al.). The main picture arising from the integration of metagenomic with recent chemical (Raman spectroscopy) and physical (Light Transmission Analysis) approaches, provided a complete picture of ecological connections, which includes different microbial degraders penetrating the parchment at different stages and triggering microbial successions according to collagen availability. The mini-review showed the parchment's degradation stages, including hydrothermal degradation of the two main collagen populations, the less stable collagen, classified as native, and the more stable, the so called stabilized collagen (Perini et al.).

The main overview from the papers published in this Research Topic is that the multi-omics revolution for microbial cultural heritage conservation is only just beginning. More exciting discoveries will occur in the future through the integration of genomics, transcriptomics, proteomics, metabolomics specialties with microbial cultivable techniques and restoration/conservation applications. Multidisciplinary approaches are therefore the key for this bright future and essential to unraveling the role that microorganisms can provide in the conservation of cultural heritage, from the perspectives of both biodeterioration and bio-restoration.

## Author Contributions

All authors listed have made a substantial, direct and intellectual contribution to the work, and approved it for publication.

## Conflict of Interest

The authors declare that the research was conducted in the absence of any commercial or financial relationships that could be construed as a potential conflict of interest.

## Publisher's Note

All claims expressed in this article are solely those of the authors and do not necessarily represent those of their affiliated organizations, or those of the publisher, the editors and the reviewers. Any product that may be evaluated in this article, or claim that may be made by its manufacturer, is not guaranteed or endorsed by the publisher.
